# Evaluation of micro-well collector for capture and analysis of aerosolized *Bacillus subtilis* spores

**DOI:** 10.1371/journal.pone.0197783

**Published:** 2018-05-30

**Authors:** Jiayang He, Nicola K. Beck, Alexandra L. Kossik, Jiawei Zhang, Edmund Seto, John Scott Meschke, Igor Novosselov

**Affiliations:** 1 University of Washington, Mechanical Engineering, Seattle, WA, United States of America; 2 University of Washington, Department of Environmental and Occupational Health Sciences, Seattle, WA, United States of America; Pacific Northwest National Laboratory, UNITED STATES

## Abstract

Bioaerosol sampling and identification are vital for the assessment and control of airborne pathogens, allergens, and toxins. In-situ analysis of chemical and biological particulate matter can significantly reduce the costs associated with sample preservation, transport, and analysis. The analysis of conventional filters is challenging, due to dilute samples in large collection regions. A low-cost cartridge for collection and analysis of aerosols is developed for use in epidemiological studies and personal exposure assessments. The cartridge collects aerosol samples in a micro-well which reduces particles losses due to the bounce and does not require any coating. The confined particle collection area (d_well_~1.4 mm) allows reducing the elution volume for subsequent analysis. The performance of the cartridge is validated in laboratory studies using aerosolized bacterial spores (*Bacillus subtilis*). Colony forming unit analysis is used for bacterial spore enumeration. Cartridge collection efficiency is evaluated by comparison with the reference filters and found to be consistent with tested flow rates. Sample recovery for the pipette elution is ~80%. Due to the high density of the collected sample, the cartridge is compatible with in-situ spectroscopic analysis and sample elution into the 10–20 μl liquid volume providing a significant increase in sample concentration for subsequent analysis.

## Introduction

Many environmental and occupational exposure studies are aimed at understanding negative effects of bioaerosols on human health. Exposure to bioaerosols is of interest in occupational settings like dairy farms, textile plants, and grain processing and the indoor air studies, e.g. [1–6]. Traditionally aerosol particles are collected onto a solid substrate or filter media, into a liquid volume, or directly deposited onto a growth media. Several techniques have been used for the collection of bioaerosols, including filter collection, centrifugal collection, electrostatic precipitation, liquid impingement, and impaction. Often, filters are used for aerosol sample collection; multiple publications describe these collection and elution procedures, review the collection efficiency of a variety of filtration media for submicron inert and biological particles. Recent reviews include performance and comparison of multiple samplers related to the collection of bioaerosols [7–10]. With respect to direct personal exposure monitoring some of these methods may not be practical; for example, filter collection and analysis is limited by high elution volumes, the form factor of the sampling setup, and high-power requirements. Due to the development in miniaturization of sampling pumps and electronics, new exposure methods are becoming more popular in epidemiological studies and air quality monitoring applications. Recent examples of miniature PM samplers are presented in [11–14] including biological aerosol collectors [15–19], real-time time PM monitoring devices [20–23], and low-cost distributed sensor networks [24–26] that are used for pollution exposure estimates.

Solid substrate collectors, such as cyclone and inertial impactors, rely on the momentum difference between airborne particulates and air molecules to collect the aerosol particles onto a solid surface. Commercially available surface collectors have been widely used by the researchers, for example, Andersen impactor directly collects the organisms onto a growth media; it has been used for the collection of many biological agents, including molds, fungal spores, and bacterial spores, e.g., [27, 28]. However, a limitation of this method is the potential to overload the collection substrate, which occurs when multiple organisms impact at the same location. Liquid collection methods originally suffered from liquid evaporation, particle bounce, and liquid loss due to aerosolization and vaporization, e.g., AGI-30 impingers [29, 30]. The BioSampler (SKC Inc., Eighty-Four, PA) was developed to increase the sampling duration and improve the collection efficiency of viable organisms. Collection through impaction and centrifugal motion allows for the particles to be collected in non-evaporating, more viscous liquids, increasing the sampling time and maintaining relatively constant physical collection efficiencies [31]. Most devices based on sampling and collection into liquid volume require significant power due to their high air flow rate; their large liquid volume sample limits their applicability in personal samplers and analysis techniques where small collection volume and direct integration with microfluidic devices is desired. Recently, several air-to-liquid interfaces for microfluidic devices have been reported [19, 32, 33]. For atmospheric science and security applications where concentrations of aerosols can be relatively low, additional particle concentrations may be required. Virtual impactors [34] and aerodynamic lens concentrators can increase the concentration by order of magnitude with minimum pressure drop while reducing the size of the particle beam [35, 36].

Primarily two types of the samplers are used to assess exposure and to characterize the environment of interest: (i) area and (ii) personal exposure samplers. Typically, area samplers are operated at high flow rates, greater than 10 standard liters per minute (slpm). For epidemiological studies, personal samplers are preferred as they allow to assess the individual exposure removing the ambiguity of temporal and spatial distribution of particulate matter. Miniaturization and integration of components for personal exposure monitors require the development and validation of novel aerosol collectors; it also presents an opportunity to improve analysis methods and enable rapid in-situ sample characterization. Recently, we presented a design methodology for a combined an aerodynamic focusing (AF) inlet and a μ-well trap compatible with a small sample elution volume and in-situ optical analysis [37]. Fluorescence techniques such as UV laser-induced fluorescence (UV-LIF) and Raman spectroscopy have been used for classification and quantitative determination of the aerosol particles [38–42]. Spectra obtained from a cluster of aerosol particles collected on the substrate provide the information about chemical compositions and biological identification of the particles. The cartridge presented in this work is designed using the previously described methodology and has been manufactured using high-volume fabrication method for application in the epidemiological research. To enable in-situ UV based analysis, the cartridge is fabricated using UV transparent materials. The dimensions of the inlet (which act as an elution port) and the micro-well (μ-well) are optimized for compatibility with the standard pipette tip and high-volume fabrication methods.

In this work, we present the performance of the low-cost μ-well aerosol collection cartridge designed for use with the personal exposure monitor. The performance of the cartridge is validated in laboratory studies using aerosolized single-organism, *Bacillus subtilis* spores based on comparison with the collection and analysis of the reference filters. Sample recovery for the pipette elution is tested for elution in the 10–20 μl liquid volume.

### Micro-well cartridge design and fabrication

**Fig 1:**
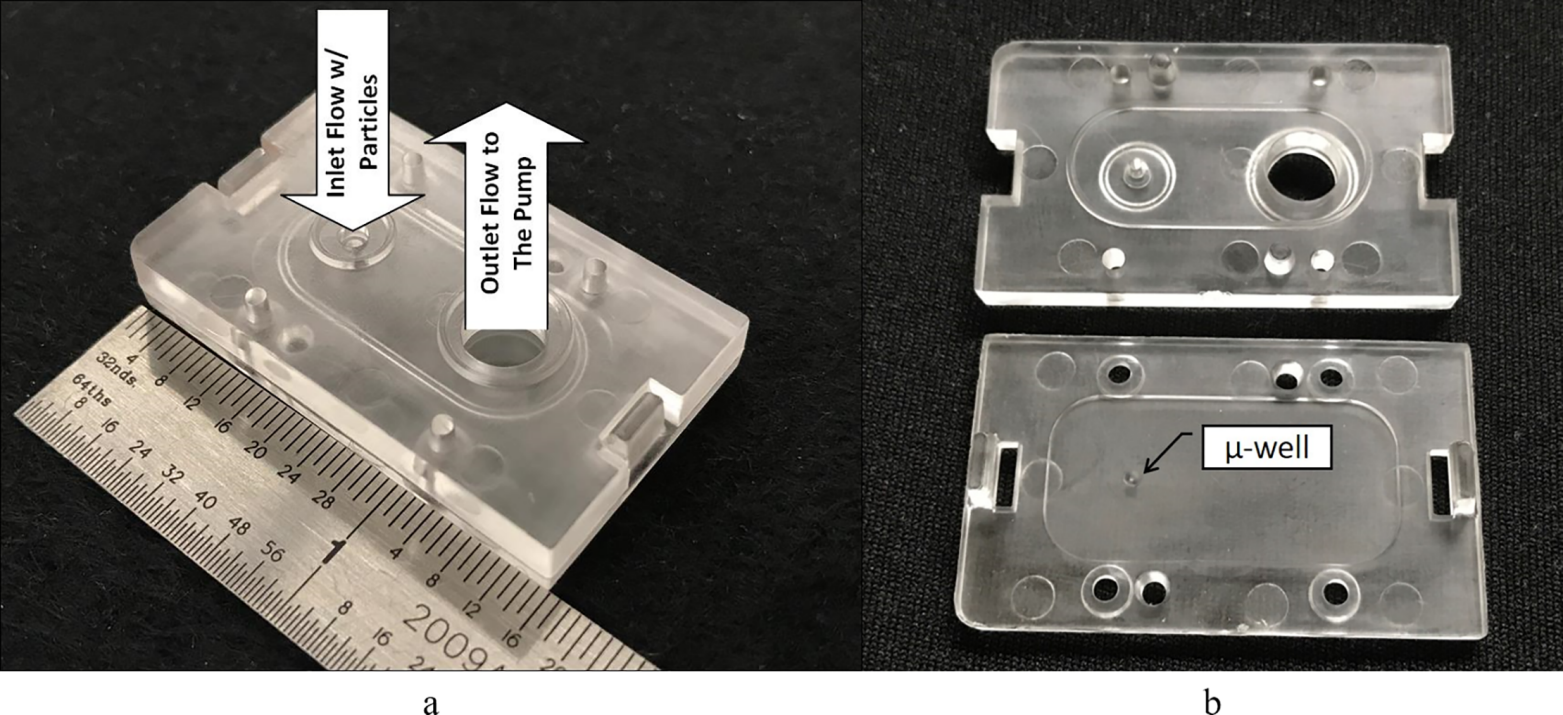
(a) The photograph of the assembled μ-well aerosol collection cartridge and (b) the cartridge in a disassembled state.

The design of the low-cost μ-well collection cartridge is based on the methodology outlined in our previous work [37]. The low-cost collection cartridge presented here consists of two injection-molded parts (see Fig 1). The top half includes an AF inlet to accelerate and focus the particles and a fluidic connection to the vacuum pump. The conical μ-well on the bottom half captures the particles into a small collection area. The range of the operating flow rate for the cartridge is 0.75–2 slpm at standard conditions (293 K, 101 kPa). The principle of operation has been previously described, typical flow field and particle behavior are shown in Figs A-B in S1 File. The Stokes number analysis is used to estimate the performance of the AF inlet, is used as the guidance of the inlet design. If the particle Stokes number of the AF inlet is smaller than the optimum Stokes number, the particles follow the flow streamlines cannot be effectively focused into the well. In contrast, if the Stokes number is near the optimum value, the particle is focused by the AF inlet and projected into the μ-well. The optimum Stokes number Stk* was selected to be 1, based on the previous studies [43, 44] and our own work based on the CFD simulations of a number of focusing inlet geometries and flow rates [37]. *Stk* ~ 0.55–0.85 have been reported for aerodynamic focusing using parallel slit geometries [35, 45]. The optimizing parameter is used as the design guidance; it does not account for variation of the Stoke number in a non-uniform velocity field, in reality, the Stokes number may vary significantly depending on the particle position, its size and density, and the device flow rate. Stk* is calculated as:

$${Stk}^{*}=\frac{\tau }{D_{c}/U}=\frac{{C_{c}\rho }_{p}d_{p}^{2}U}{18\eta D_{c}}≅1$$ (1)

where *C*_*c*_ is the Cunningham slip correction factor for the particle, *ρ*_*p*_ is the particle density, *d*_*p*_ is the particle diameter, *U* is the area-averaged flow axial velocity magnitude at a well upstream location, *η* is the gas viscosity, and *D*_*c*_ is the characteristic dimension, which, in this case, is the diameter of the nozzle.

The geometry of the inlet also satisfies the practical considerations of the compatibility with a standard pipette tip for the sample elution. The diameter of the inlet is chosen based on the calculation of the Stokes number, as well as compatibility with the standard pipette tip. The angle of the μ-well cone of 35 degrees is selected to reduce the effect of particle bounce at the collection location. Our previous study shows that larger angles are more effective for the mitigation of particle bounce; however, they are less efficient for the collection of smaller particles. Fig 1A shows the photograph of the cartridge with arrows indicating the flow direction. Fig 1B shows the cartridge in a disassembled state to reveal the μ-well on the bottom half of the cartridge. The design drawings are shown in Fig C in S1 File.

The μ-well cartridges are fabricated using the injection molding technique. The cost of each cartridge is under $5 for low production run (n = 500) with the aluminum mold used. Each collector is assembled from two molded parts, with a gasket between them. A 0.5 mm thick silicone gasket is used to provide the proper nozzle-to-plate distance; the gasket thickness can be changed to vary the distance between the nozzle and the μ-well, which, in turn, changes the cartridge collection characteristics. The seal between the nozzle and the impaction plate is achieved by applying compression force from the two snap-on tabs. All collectors are checked for a vacuum seal before conducting the experiments. The dimensions of a collector are about 40 mm × 23 mm × 5 mm. Other critical dimensions of the AF inlet and the μ-well are shown in Fig C in S1 File. Both the inlet and outlet of the μ-well cartridge are located on the same side of the geometry to permit the easy integration of the collector array and the vacuum manifold, as well as to ensure proper sealing to avoid sample contamination during storage and handling. The overall quality of the injection-molded μ-well cartridge was found to be very good: the production parts did not have the defects often associated with injection molding, such as sinking, burning, flashing, or short shots. All tested collector assemblies fit together well and provided a good seal for all tested operating pressures. The pressure drop through the collectors was found to be consistent for any given flow rate within the accuracy of the measuring instruments (~5%).

### Experimental methods

**Fig 2:**
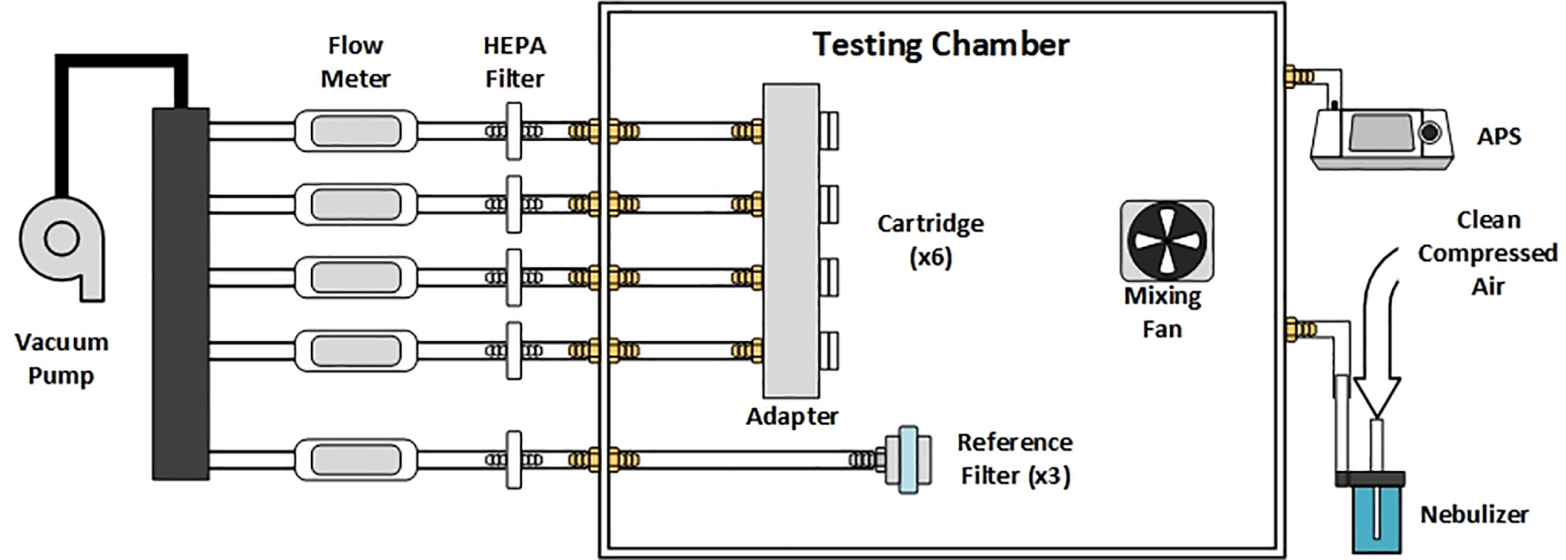
The configuration of the aerosol chamber experiment.

The collection efficiency of the cartridge was evaluated by collecting aerosolized single-organisms *B*. *subtilis* (ATCC® 31578™ Strain Designation: RUB331) in comparison with the collection of the reference filters. The collection experiments were performed in a custom 0.3 m^3^ stainless steel, well mixed aerosol chamber (see Fig 2) for a range of flow rates of 0.75–2.0 slpm. The large volume of the chamber with mixing fans provides well-mixed conditions and allows for evaluation multiple collectors simultaneously. The aerosol concentration in the chamber was found to be spatially uniformed with the operation of the mixing fans. Typically an array of six collectors and three reference filters were used in the experiments. The collectors are fluidically connected to the mass flowmeters (HONEYWELL, Morristown, NJ, AWM5102VN) via an adapter. The reference filter housed in the filter housing, similarly connected house vacuum through the same type flow meters. Three reference filters (Spectrum Poretics® polycarbonate membrane filters, 47mm, 0.6 μm pore sizes) in open face aerosol filter holders (EDM Millipore, Billerica, MA, model XX5004710) collect particles at 1 slpm in each experiment. All flowmeters are located outside the chamber allowing for individual control of the flow rate during the experiment. The flowmeters are calibrated before and after each experiment using Gillibrator (Sensidyne, Clearwater, FL, Gilibrator 2). All experiments were performed at the room temperature of 20–25°C and relative humidity in the chamber of 40–60%. Operating flow rates for the cartridges were 0.75, 1, 1.5, and 2 slpm.

A *B*. *subtilis* spore stock was prepared by initially growing the *B*. *subtilis* overnight in Luria-Bertani Broth (LB) at 37° C in an incubator shaker. The overnight culture was spread on AK#2 sporulating agar, and the plates were incubated for 48 hours at 37° C. The growth was scraped off the plates, and the stock was stored in water at 4° C. The stock was purified by centrifuging at 10,000 G for 10 minutes and washed in cold, sterile water; this procedure was repeated three times. Subsequently, the pellet was shaken overnight at 125 rpm and 4° C, centrifuged for 20 minutes at 20,000 g and resuspended in the new cold, sterile water. This procedure was repeated for several days until the pellet formed a homogeneous layer. The stock was checked for purity with microscopy using a malachite green spore stain. The purified stock was stored at 4° C.

**Fig 3:**
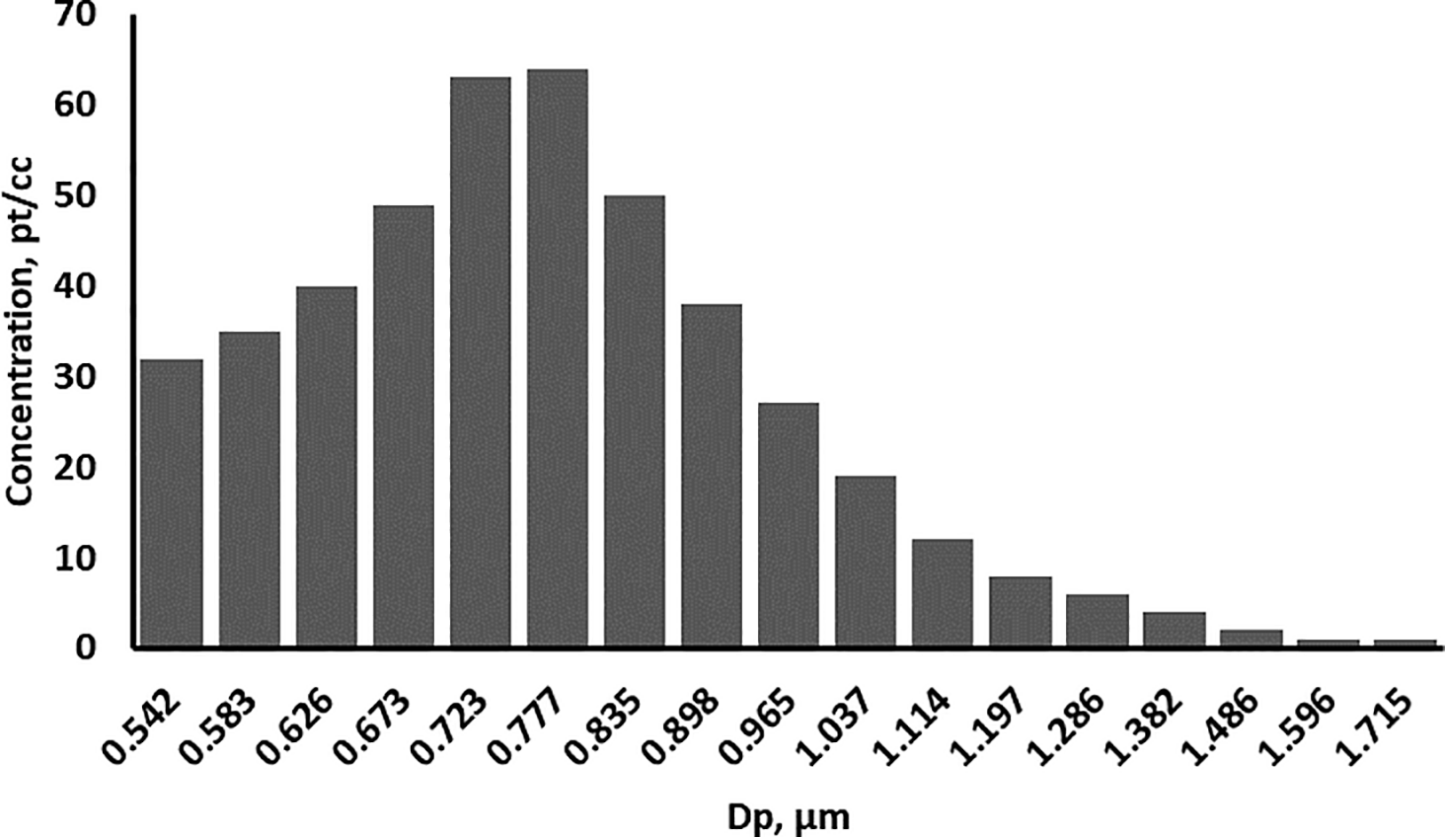
The size distribution of airborne particles.

The prepared bacterial spore suspension was diluted 100x in the distilled water right before the experiment. During the experiment, 4 mL of prepared solution was nebulized from the liquid suspension with 20 psi clean air using a Lovelace nebulizer (In-Tox Products, Moriarty, NM). An aerodynamic particle sizer (APS 3321, TSI, Shoreview, MN) was used to verify the particle size and monitor the particle concentration in the chamber. The aerodynamic diameter of the *B*. *subtilis* spores was measured by the APS to be about 0.75 μm. APS measurements also confirm that no significant particle agglomeration occurs inside the chamber during the experiment. Fig 3 shows a typical particle size distribution during the experiments.

Following the collection experiment, two elution steps were performed to remove *B*. *subtilis* from the collectors. The efficiency of each elution procedure was quantified. The first elution step is used quantify the sample collected in the in μ-well and in the close vicinity (1-2mm) around the well. The second step is used to elute all the particulate matter collected elsewhere inside the cartridge during the experiment; these include the particles lost on the upper collector part, the gasket, and around the outlet of the cartridge. For the first elution, small liquid aliquots were used: 2 x 10 μl of Phosphate Buffered Saline (PBS) with 0.05% Tween 20. Each 10 μl was added to the collector and immediately pipetted up/down 10 times, after which the droplet was placed in a microcentrifuge tube containing 980 μl of PBS and vortexed for 1 minute before diluting in PBS. A second elution consists of disassembling and submerging the entire cartridge in a centrifuge tube with 10 ml of PBS with 0.05% Tween. The centrifuge tube undergoes 10 minutes of shaking on a shaker table, followed by 5 minutes of sonication in a 60 Watts ultrasonic cleaner bath (model #gb928). After the collection, the reference filters were submerged in 10 ml of PBS with 0.05% Tween and underwent a 10-min shaking period, followed by 5 minutes of sonication. While sonication is a recognized method of spore recovery [46, 47], experiments plating dilutions of the *B*. *subtilis* spore stock show that the sonication step may reduce the spore stock titer by up to 30%.

The elution efficiency and the collection efficiency were calculated by comparing the *B*. *subtilis* Colony forming unit (CFU) for the cartridge and the reference filter elution, the flow rate adjustments were made to account for differences in sampling rates. The collection efficiencies are calculated based on the results of at least 6 data points from three runs for each flow condition. Mean, and one σ error bars are presented in the collection efficiency plot. CFU was quantified on nutrient agar plates by plating 100 μl of the relevant dilutions in duplicate and incubating at 37° C overnight, after which the CFU captured at each flow rate were enumerated. The undiluted sample and a 10x dilution were plated for the first elution of the cartridge; the undiluted samples were plated for the second elution. Both the undiluted and the 10x dilution were plated for the reference filters. In the analysis, the CFU counts were adjusted for the difference in elution volumes between the first, the most concentrated eluent, the reference filters, and the second elution step.

## Results and discussion

### Pressure drop

**Fig 4:**
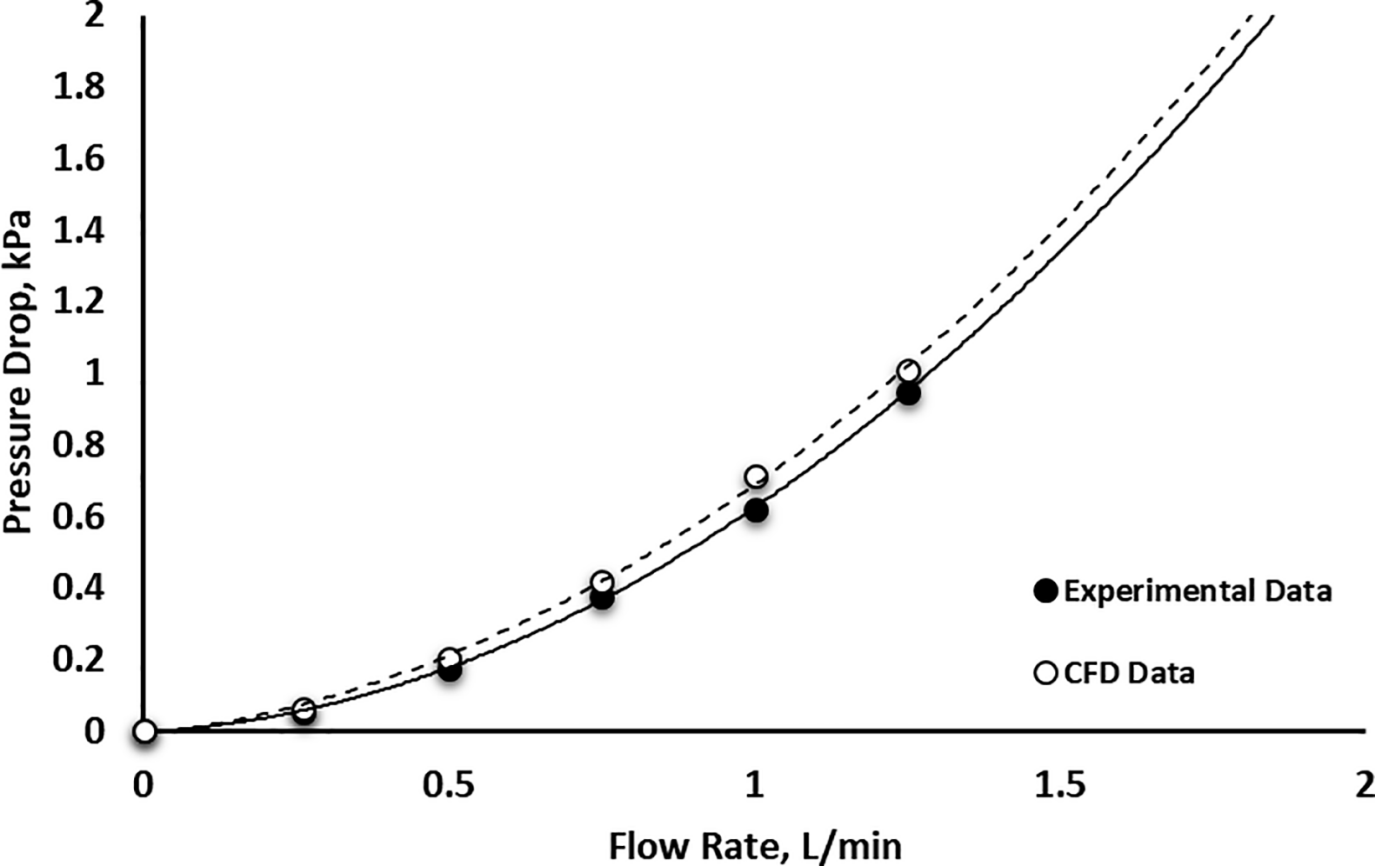
The measured pressure drop of the collection cartridge at different flow rates.

To estimate the power consumption and to aid with the selection of the pump for the personal exposure monitors, we evaluated the cartridge for pressure drop as a function of the flow rates. Fig **4** shows the measured pressure drop for the cartridge, as well as the pressure drop predicted by the computational fluid dynamics (CFD). The pressure drop was measured using a Magnehelic gauge (Series 2100 Magnehelic® Differential Pressure Gage, Dwyer, Michigan City, IN). Three measurements are performed for each operating condition; the measurement error did not exceed 5%. The pressure drop in the cartridge was mostly due to the losses in the nozzle and the abrupt change of the flow direction at the impaction plate. The experimental pressure drop of the low-cost cartridge was compared to the results of the computational fluid dynamics study performed during the design stage [37]. The results show excellent agreement between the computational and experimental pressure drop measurements. The cartridges are evaluated for potential failure due to the collection overloading. The pressure drop of an overloaded cartridge was consistent with the blank one; this indicates that loading of the collector does not increase the pressure drop, unlike filter collection [48].

### Bioaerosol collection efficiencies and analysis

**Fig 5:**
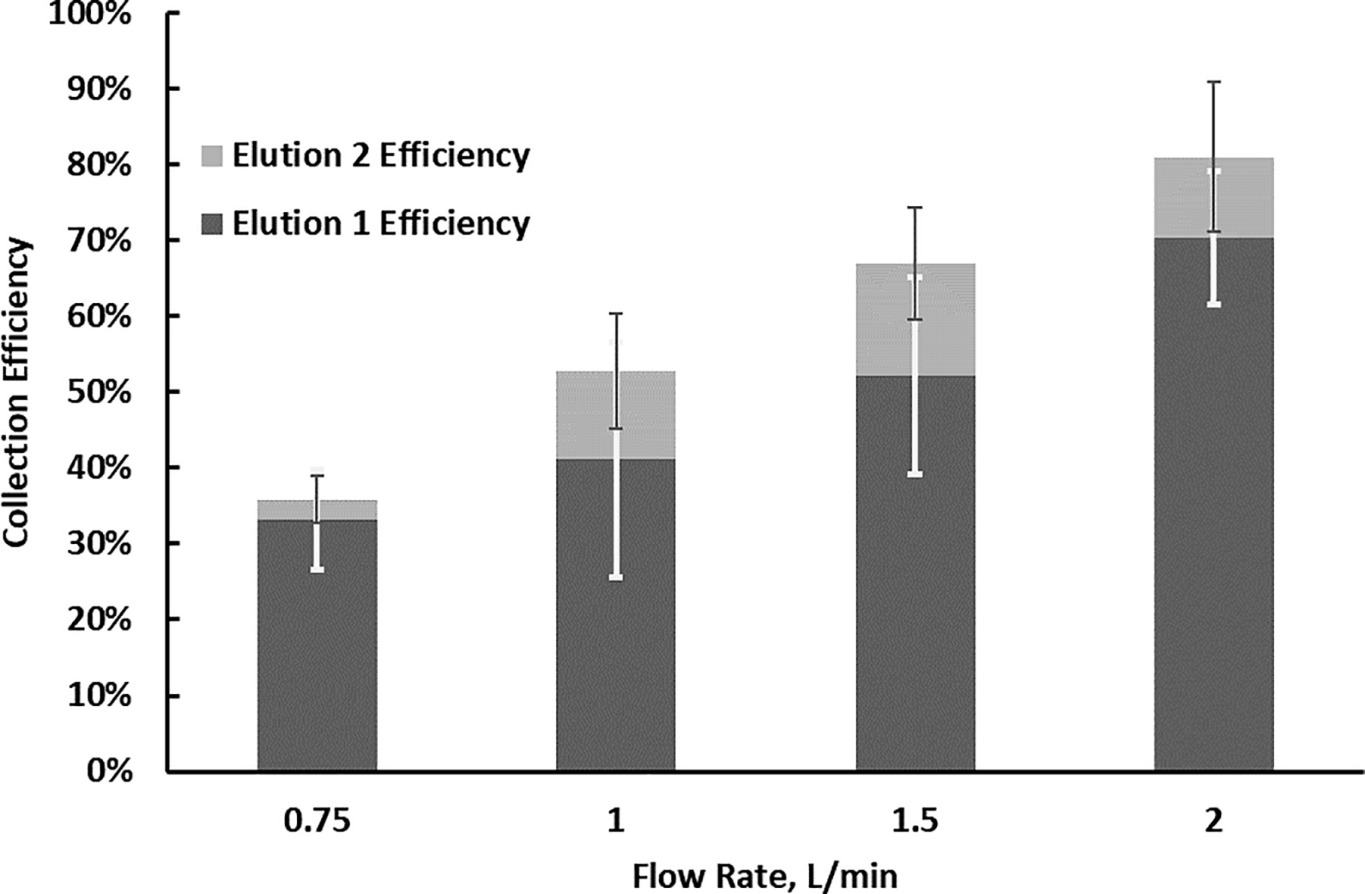
*B*. *subtilis* collection efficiencies in μ-well inertial impactors as a function of the collector flow rate.

The collection efficiencies are presented in Fig **5**. The results show that the collection efficiency for the *B*. *subtilis* spore increases with the flow rate. At a flow rate of 0.75 slpm, the cartridge collects about 35% of the *B*. *subtilis* spores as compared to the number collected by the reference filter. The maximum collection efficiency observed is about 80% at the highest flow rate tested (2 slpm). The results are consistent with the inertial mechanism of particle capture. The particle with the lower Stokes number, associated with the low velocities in the focusing inlet, can not reach the μ-well wall [37]. Higher sampling rates are desired to improve the capture efficiency of single-organism bioaerosols. Unlike conventional impactors without coating, no significant collection efficiency drop associated with particle bounce is observed in the collection of *B*. *subtilis* as the majority of the spores were recovered from the direct well elution (elution 1); less than 25% was recovered during the second elution when the cartridge was disassembled and sonicated. The limited particle loss from the targeted collection area can be attributed to the μ-well angle, which redirects the bounce toward the bottom of the well and greatly eliminates the particles from re-entering the major flow. Though the preliminary results from the chamber experiments and the pilot indoor and outdoor sampling show significant accumulation of sample in the μ-well (see Fig D in S1 File), the bounce mechanisms for biological and other particles needs further investigation. A single pipette elution extracts more than 75% of the *B*. *subtilis* collected in the μ-well cartridge for all tested conditions. The small elution volume is beneficial for bioaerosol sampling providing two to three order magnitude preconcentration of the analysis volume. We tested the cartridge compatibility with the fluorescence measurement using the fluorescent PSL microspheres. The method and the setup used for the measurement is described in the SI document. The spectrum from the in-situ fluorescence measurement on the cartridge shows a good agreement with the spectrum acquired from the liquid sample (see Fig E in S1 File).

## Conclusions and discussions

Performance of a low-cost μ-well aerosol cartridge is evaluated for collection and analysis of single-organism *B*. *subtilis* spores. The design of the device is based on practical considerations for sample analysis; the inlet dimensions are compatible with the pipette sample elution and integration with personal exposure monitors. The cartridge collects highly concentrated particle samples in a 1 mm diameter spot. The sample is retained in the well because the particle bounce is redirected toward the center of the well, increasing the sample collection density. Collection efficiency is consistent for each flow condition. Sample recovery for the pipette elution is greater than 75%. The elution volume used to recover the collected sample from the cartridge is in the range of 10–20 μl, which provides a high preconcentration of the aerosol sample for liquid assays, based on the cartridge geometry the volume can be further reduced if the procedure is automated or microfluidic analysis is desired.

While in this manuscript we use the standard laboratory analysis method, the optically transparent collection substrate and well-defined collection region allow for the in-situ optical analysis of the collected aerosols. This in-situ spectroscopic analysis may provide a non-destructive orthogonal data such as fluorescent measurement and a significant reduction in analysis cost for monitoring exposure to chemical and biological aerosols, toxic compounds in indoor environment and other applications. In our preliminary analysis (see Supplemental Materials), we demonstrated the in-situ detection of fluorescent 2 μm PSL spheres collected in the aerosol chamber experiments. The optimization of the optical cell geometry is required to minimize the scatter and reflection from the cartridge surfaces. One promising approach is to use fiber-optic excitation and collection of the signal in a backscatter probe configuration; this would significantly minimize the sensor footprint. Multiple reviews in the topic of bio and chemical detection using fiber-optic sensor exist, e.g., [49, 50]. Raman backscatter probes are well-developed, e.g., [51–53] and can be readily used with the cartridge without significant hardware modification. Fiber-optic probes for surface-enhanced Raman [54] and anti-stokes Raman [55] have been demonstrated for analysis for detection of bioaerosols. Sample collected in the μ-well can be an analysis based on native fluorescence of the bioaerosols such as UV LIF [56, 57], though the application of this technique using fiber-optic bundles needs further development.

## Supporting information

S1 File Fig A. Gas streamlines; The colormap represents the air velocity magnitude in m/s (Nozzle diameter: 0.8 mm; Re = 1815).
Fig B. Trajectories of particles in different impactors at 1 slpm. (a) particle trajectories in the AF μ-well impactor. The dashed area is expanded in (b) to show the details; (c) detailed view of the particle trajectories for the μ-well impactor with a straight nozzle. Particle size: red line– 3 μm, green line– 2 μm, blue line– 1 μm (Nozzle Diameter: 0.8mm; Re = 1815).
Fig C. (a) The μ-well aerosol collection cartridge and (b) the dimensions of the cartridge; (c) the critical dimensions of the AF inlet and the μ-well (unit: mm).
Fig D. Microscopic image of PM collected in the cartridges during the one-week usability study.
Fig E. (a) The cartridge fluorescence measurement setup and (b) the fluorescent PSL particle collection site; (c) the fluorescence spectrum for the liquid and solid sample.
(DOCX)

## Abbreviations

d_well_: Diameter of the μ-well
C_c_: Cunningham correction factor
D_c_: characteristic dimension of the impactor (m)
Q: volumetric flowrate (m^3^/s)
Stk*: optimum Stokes number
U: area-average flow velocity (m/s)
d_p_: particle diameter (m)
τ: relaxation time (s)
η: dynamic viscosity (Pa·s)
ρ_p_: particle density (kg/ m^3^)

## References

[1] O'Hara RE, Rubin R. Reducing bioaerosol dispersion from wastewater treatment and its land application: a review and analysis. Journal of environmental health. 2005;68(2):24 16220719

[2] Daisey JM, Angell WJ, Apte MG. Indoor air quality, ventilation and health symptoms in schools: an analysis of existing information. Indoor air. 2003;13(1):53–64. 12608926 10.1034/j.1600-0668.2003.00153.x

[3] Godwin C, Batterman S. Indoor air quality in Michigan schools. Indoor air. 2007;17(2):109–21. doi: 10.1111/j.1600-0668.2006.00459.x 17391233 10.1111/j.1600-0668.2006.00459.x

[4] Nazaroff WW. Indoor bioaerosol dynamics. Indoor Air. 2016;26(1):61–78. doi: 10.1111/ina.12174 25483392 10.1111/ina.12174 PMC7165847

[5] Triadó‐Margarit X, Veillette M, Duchaine C, Talbot M, Amato F, Minguillón MC, et al Bioaerosols in the Barcelona subway system. Indoor air. 2017;27(3):564–75. doi: 10.1111/ina.12343 27687789 10.1111/ina.12343

[6] Heutte N, André V, Arvis CD, Bouchart V, Lemarié F, Legendre P, et al Assessment of multi-contaminant exposure in a cancer treatment center: a 2-year monitoring of molds, mycotoxins, endotoxins, and glucans in bioaerosols. Environmental monitoring and assessment. 2017;189(1):31 doi: 10.1007/s10661-016-5751-z 28012082 10.1007/s10661-016-5751-z

[7] Reponen T. Sampling for Microbial Determinations Exposure to Microbiological Agents in Indoor and Occupational Environments: Springer; 2017 p. 85–96.

[8] Kesavan J, Sagripanti J-L. Evaluation criteria for bioaerosol samplers. Environmental Science: Processes & Impacts. 2015;17(3):638–45. 10.1039/c4em00510d 25631321

[9] Wang C-H, Chen BT, Han B-C, Liu AC-Y, Hung P-C, Chen C-Y, et al Field evaluation of personal sampling methods for multiple bioaerosols. PloS one. 2015;10(3):e0120308 doi: 10.1371/journal.pone.0120308 25799419 10.1371/journal.pone.0120308 PMC4370695

[10] Grinshpun SA, Buttner MP, Mainelis G, Willeke K. Sampling for airborne microorganisms Manual of Environmental Microbiology, Fourth Edition: American Society of Microbiology; 2016 p. 3.2. -1–3.2. -17.

[11] Volckens J, Quinn C, Leith D, Mehaffy J, Henry C, Miller‐Lionberg D. Development and evaluation of an ultrasonic personal aerosol sampler. Indoor air. 2017;27(2):409–16. doi: 10.1111/ina.12318 27354176 10.1111/ina.12318 PMC5199626

[12] Koehler KA, Peters TM. New methods for personal exposure monitoring for airborne particles. Current environmental health reports. 2015;2(4):399–411. 10.1007/s40572-015-0070-z PMC4807653 26385477

[13] Chartier R, Phillips M, Mosquin P, Elledge M, Bronstein K, Nandasena S, et al A comparative study of human exposures to household air pollution from commonly used cookstoves in Sri Lanka. Indoor air. 2017;27(1):147–59. doi: 10.1111/ina.12281 26797964 10.1111/ina.12281

[14] Cai J, Yan B, Ross J, Zhang D, Kinney PL, Perzanowski MS, et al Validation of MicroAeth® as a black carbon monitor for fixed-site measurement and optimization for personal exposure characterization. Aerosol and air quality research. 2014;14(1):1 doi: 10.4209/aaqr.2013.03.0088 25419215 10.4209/aaqr.2013.03.0088 PMC4240508

[15] Roux J, Sarda-Estève R, Delapierre G, Nadal M, Bossuet C, Olmedo L. Development of a new portable air sampler based on electrostatic precipitation. Environmental Science and Pollution Research. 2016;23(9):8175–83. doi: 10.1007/s11356-015-5522-3 26452658 10.1007/s11356-015-5522-3

[16] Han TT, Thomas NM, Mainelis G. Design and development of a self-contained personal electrostatic bioaerosol sampler (PEBS) with a wire-to-wire charger. Aerosol Science and Technology. 2017;51(8):903–15.

[17] Foat T, Sellors W, Walker M, Rachwal P, Jones J, Despeyroux D, et al A prototype personal aerosol sampler based on electrostatic precipitation and electrowetting-on-dielectric actuation of droplets. Journal of Aerosol Science. 2016;95:43–53.

[18] Ariessohn PC, Novosselov IV. Aerosol Collection and Microdroplet Delivery for Analysis. Google Patents; 2010.

[19] Novosselov IV, Gorder RA, Van Amberg JA, Ariessohn PC. Design and Performance of a Low-Cost Micro-Channel Aerosol Collector. Aerosol Science and Technology. 2014;48(8):822–30. doi: 10.1080/02786826.2014.932895

[20] Dacunto PJ, Klepeis NE, Cheng K-C, Acevedo-Bolton V, Jiang R-T, Repace JL, et al Determining PM 2.5 calibration curves for a low-cost particle monitor: common indoor residential aerosols. Environmental Science: Processes & Impacts. 2015;17(11):1959–66. 10.1039/c5em00365b 26487426

[21] Njalsson T, Novosselov I. Design and Optimization of a Compact Low-cost Optical Particle Sizer. Journal of Aerosol Science. 2018. 10.1016/j.jaerosci.2018.01.003 PMC6159267 30270936

[22] Austin E, Novosselov I, Seto E, Yost MG. Laboratory Evaluation of the Shinyei PPD42NS Low-Cost Particulate Matter Sensor. PloS one. 2015;10(9):e0137789 doi: 10.1371/journal.pone.0137789 26367264 10.1371/journal.pone.0137789 PMC4569398

[23] Dons E, Laeremans M, Orjuela JP, Avila-Palencia I, Carrasco-Turigas Gr, Cole-Hunter T, et al Wearable sensors for personal monitoring and estimation of inhaled traffic-related air pollution: evaluation of methods. Environmental science & technology. 2017;51(3):1859–67. 28080048 10.1021/acs.est.6b05782

[24] Gao M, Cao J, Seto E. A distributed network of low-cost continuous reading sensors to measure spatiotemporal variations of PM2. 5 in Xi'an, China. Environmental pollution. 2015;199:56–65. doi: 10.1016/j.envpol.2015.01.013 25618367 10.1016/j.envpol.2015.01.013

[25] Seto E, Austin E, Novosselov I, Yost M, editors. Use of low-cost particle monitors to calibrate traffic-related air pollutant models in urban areas. 7th International Congress on Environmental Modelling and Software, iEMSs 2014; 2014: International Environmental Modelling and Software Society.

[26] Johnson KK, Bergin MH, Russell AG, Hagler GS. Using low cost sensors to measure ambient particulate matter concentrations and on-road emissions factors. Atmospheric Measurement Techniques Discussions. 2016:1–22.

[27] Wang Z, Reponen T, A. Grinshpun S, L. Górny R, Willeke K. Effect of sampling time and air humidity on the bioefficiency of filter samplers for bioaerosol collection. Journal of Aerosol Science. 2001;32(5):661–74. http://dx.doi.org/10.1016/S0021-8502(00)00108-7.

[28] Radosevich J, Wilson W, Shinn J, DeSantis T, Andersen G. Development of a high‐volume aerosol collection system for the identification of air‐borne micro‐organisms. Letters in applied microbiology. 2002;34(3):162–7. 11874535 10.1046/j.1472-765x.2002.01048.x

[29] Willeke K, Lin XJ, Grinshpun SA. Improved aerosol collection by combined impaction and centrifugal motion. Aerosol Science and Technology. 1998;28(5):439–56. doi: 10.1080/02786829808965536 PubMed PMID: WOS:000073164900004.

[30] Agranovski IE, Safatov AS, Borodulin AI, Pyankov OV, Petrishchenko VA, Sergeev AN, et al New personal sampler for viable airborne viruses: feasibility study. Journal of Aerosol Science. 2005;36(5):609–17. doi: 10.1016/j.jaerosci.2004.11.014

[31] Lin X, Willeke K, Ulevicius V, Grinshpun S. Effect of Sampling Time on the Collection Efficiency of All-Glass Impingers. American Industrial Hygiene Association Journal. 1997;58(7):480–8. doi: 10.1080/15428119791012577

[32] Ariessohn PC, Novosselov IV. Aerosol Collection and Microdroplet Delivery for Analysis. Google Patents; 2009.

[33] Damit B. Droplet-based microfluidics detector for bioaerosol detection. Aerosol Science and Technology. 2017;51(4):488–500.

[34] Marple VA, Chien CM. Virtual impactors: a theoretical study. Environmental science & technology. 1980;14(8):976–85. 22296546 10.1021/es60168a019

[35] Novosselov IV, Ariessohn PC. Rectangular slit atmospheric pressure aerodynamic lens aerosol concentrator. Aerosol Science and Technology. 2014;48(2):163–72.

[36] Ariessohn PC, Novosselov IV. Skimmer for concentrating an aerosol. US Patent 7875095; 2011.

[37] He J, Novosselov IV. Design and evaluation of an aerodynamic focusing micro-well aerosol collector. Aerosol Science and Technology. 2017;(just-accepted). 10.1080/02786826.2017.1329515 PMC6368264 30739977

[38] Pan Y-L. Detection and characterization of biological and other organic-carbon aerosol particles in atmosphere using fluorescence. Journal of Quantitative Spectroscopy and Radiative Transfer. 2015;150:12–35. doi: 10.1016/j.jqsrt.2014.06.007

[39] Sengupta A, Laucks ML, Dildine N, Drapala E, Davis EJ. Bioaerosol characterization by surface-enhanced Raman spectroscopy (SERS). Journal of Aerosol Science. 2005;36:651–64. doi: 10.1016/j.jaerosci.2004.11.001

[40] Dippel B, Heintzenberg J. Soot characterization in atmospheric particles from different sources by NIR FT Raman spectroscopy. Journal of Aerosol Science. 1999;30:S907–S8. doi: 10.1016/S0021-8502(99)80464-9

[41] Huffman J, Santarpia J. Online Techniques for Quantification and Characterization of Biological Aerosols. Microbiology of Aerosols. 2017:83–114.

[42] Campbell SD, Tremblay DP, Daver F, Cousins D, editors. Multiwavelength bioaerosol sensor performance modeling Optically Based Materials and Optically Based Biological and Chemical Sensing for Defence II; 2005: International Society for Optics and Photonics.

[43] Deng R, Zhang X, Smith KA, Wormhoudt J, Lewis DK, Freedman A. Focusing Particles with Diameters of 1 to 10 Microns into Beams at Atmospheric Pressure. Aerosol Science and Technology. 2008;42(11):899–915. doi: 10.1080/02786820802360674

[44] Zhang X, Smith KA, Worsnop DR, Jimenez J, Jayne JT, Kolb CE. A Numerical Characterization of Particle Beam Collimation by an Aerodynamic Lens-Nozzle System: Part I. An Individual Lens or Nozzle. Aerosol Science and Technology. 2002;36(5):617–31. doi: 10.1080/02786820252883856

[45] Goo J. Numerical simulation of aerosol concentration at atmospheric pressure by a cascade of aerodynamic slit lenses. Journal of aerosol science. 2002;33(11):1493–507.

[46] Al LRe. Swab Materials and Bacillus anthracis Spore Recovery from Nonporous Surfaces—Volume 10, Number 6—6 2004—Emerging Infectious Disease journal—CDC. doi: 10.3201/eid1006.030716 15207053 10.3201/eid1006.030716 PMC3323178

[47] Development OoR. Literature Review of Protocols for Processing Soils Contaminated with Bacillus anthracis Spores.

[48] Payet S, Boulaud D, Madelaine G, Renoux A. Penetration and pressure drop of a HEPA filter during loading with submicron liquid particles. Journal of Aerosol Science. 1992;23(7):723–35. http://dx.doi.org/10.1016/0021-8502(92)90039-X.

[49] Pospíšilová M, Kuncová G, Trögl J. Fiber-optic chemical sensors and fiber-optic bio-sensors. Sensors. 2015;15(10):25208–59. doi: 10.3390/s151025208 26437407 10.3390/s151025208 PMC4634516

[50] Wolfbeis OS. Fiber-optic chemical sensors and biosensors. Anal Chem. 2008;80(12):4269–83. doi: 10.1021/ac800473b 18462008 10.1021/ac800473b

[51] Kudelski A. Analytical applications of Raman spectroscopy. Talanta. 2008;76(1):1–8. doi: 10.1016/j.talanta.2008.02.042 18585231 10.1016/j.talanta.2008.02.042

[52] Marquardt BJ, Le T, Burgess LW, editors. Demonstration of a high-precision optical probe for effective sampling of solids by Raman spectroscopy Raman Spectroscopy and Light Scattering Technologies in Materials Science; 2001: International Society for Optics and Photonics.

[53] Farquharson S, Grigely L, Khitrov V, Smith W, Sperry JF, Fenerty G. Detecting Bacillus cereus spores on a mail sorting system using Raman spectroscopy. Journal of Raman spectroscopy. 2004;35(1):82–6.

[54] Stiles PL, Dieringer JA, Shah NC, Van Duyne RP. Surface-enhanced Raman spectroscopy. Annu Rev Anal Chem. 2008;1:601–26. 10.1146/annurev.anchem.1.031207.112814 20636091

[55] Petrov GI, Yakovlev VV, Sokolov AV, Scully MO. Detection of Bacillus subtilis spores in water by means of broadband coherent anti-Stokes Raman spectroscopy. Optics Express. 2005;13(23):9537–42. 19503156 10.1364/opex.13.009537

[56] Sivaprakasam V, Huston AL, Scotto C, Eversole JD. Multiple UV wavelength excitation and fluorescence of bioaerosols. Optics express. 2004;12(19):4457–66. 19483996 10.1364/opex.12.004457

[57] Eversole J, Cary W, Scotto C, Pierson R, Spence M, Campillo A. Continuous bioaerosol monitoring using UV excitation fluorescence: Outdoor test results. Field Analytical Chemistry & Technology. 2001;5(4):205–12.

