# Supplementary material for: Evaluation of micro-well collector for capture and analysis of aerosolized Bacillus subtilis spores
Source: PLoS One. 2018 May 30;13(5):e0197783. doi: 10.1371/journal.pone.0197783 (PMC5976188; doi:10.1371/journal.pone.0197783)
Supplement: S1 File — Fig A. Gas streamlines; The colormap represents the air velocity magnitude in m/s (Nozzle diameter: 0.8 mm; Re = 1815). Fig B. Trajectories of particles in different impactors at 1 slpm. (a) particle trajectories in the AF μ-well impactor. The dashed area is expanded in (b) to show the details; (c) detailed view of the particle trajectories for the μ-well impactor with a straight nozzle. Particle size: red line– 3 μm, green line– 2 μm, blue line– 1 μm (Nozzle Diameter: 0.8mm; Re = 1815). Fig C. (a) The μ-well aerosol collection cartridge and (b) the dimensions of the cartridge; (c) the critical dimensions of the AF inlet and the μ-well (unit: mm). Fig D. Microscopic image of PM collected in the cartridges during the one-week usability study. Fig E. (a) The cartridge fluorescence measurement setup and (b) the fluorescent PSL particle collection site; (c) the fluorescence spectrum for the liquid and solid sample. (DOCX) [file pone.0197783.s001.docx]

Supplemental Information: Evaluation of Micro-Well Collector for Capture and Analysis of Aerosolized Bacillus Subtilis Spores

The principle of operation is shown below by the result of the CFD model [1]. The µ-well impactor is coupled with an AF inlet to improve the collection and concentration of the collected sample in the small region. Focusing particles into the centerline of the jet allows the particles to accelerate to a higher speed, which enhances particle impaction or trapping in the recirculation zone inside the µ-well. Figure A shows a typical result for the air streamlines in the impactor. The colormap represents the air velocity magnitude in m/s. Figure B shows the trajectories of particles released from the inlet for the different impactors. The AF inlet focused particles into the center of the nozzle, which increases their speed and the collection probability. However, some larger particles (D_p_ >2 µm) injected near the wall can be lost on the forward-facing step of the AF inlet reducing the collection efficiency.

|  |  |
| --- | --- |
| 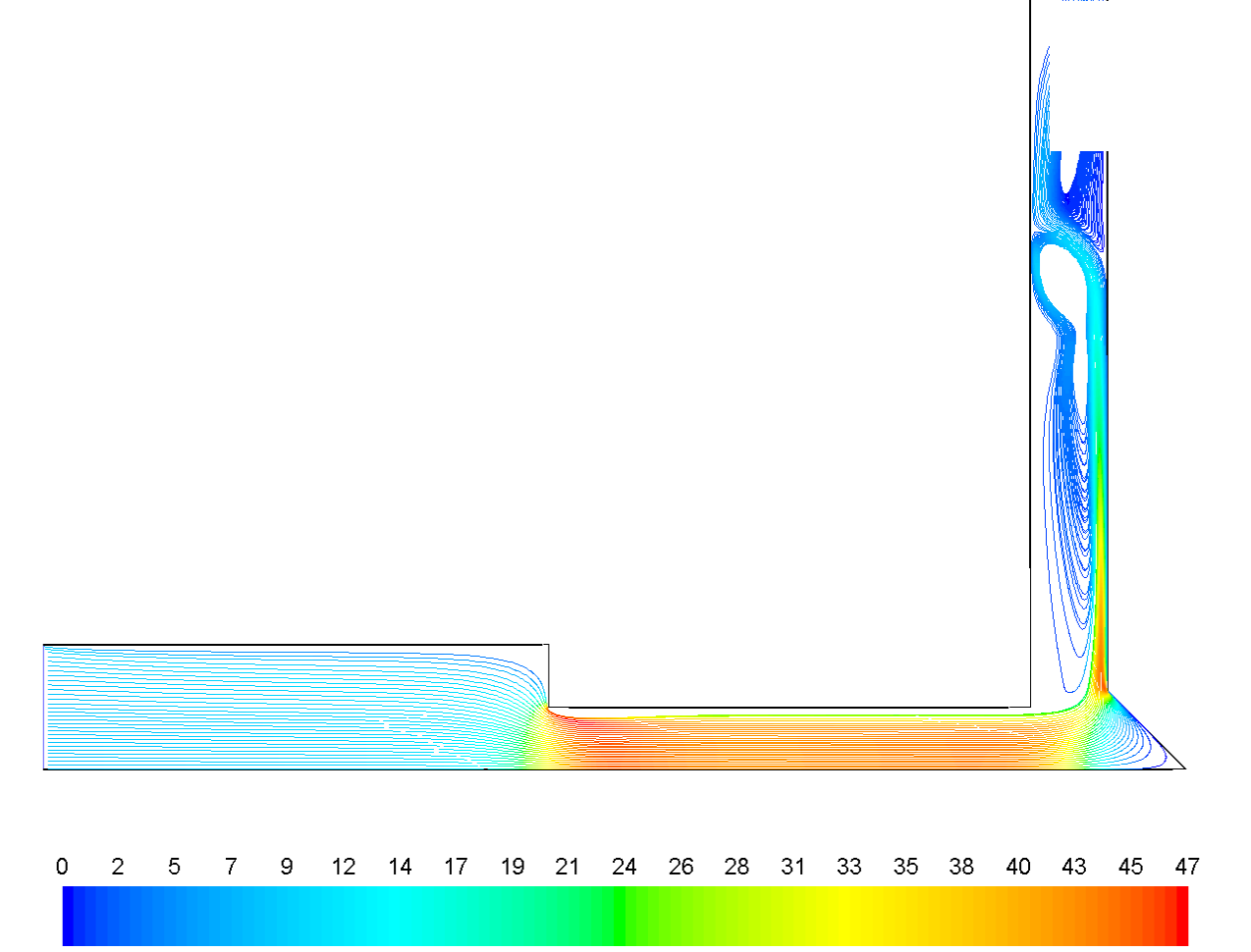 | |
| Figure A. Gas streamlines; The colormap represents the air velocity magnitude in m/s (Nozzle diameter: 0.8 mm; Re=1815), from [1] | |

| 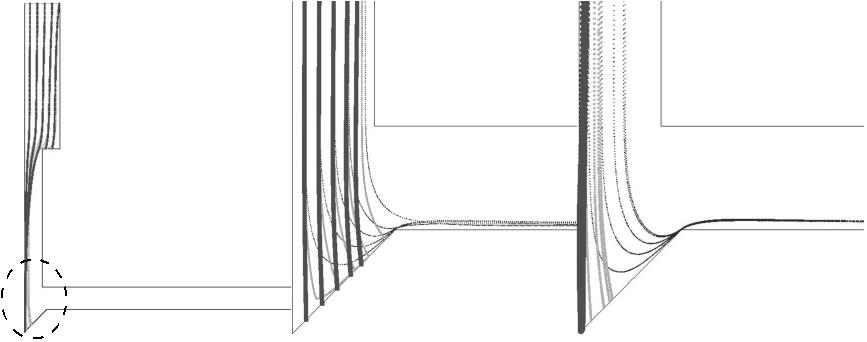 | 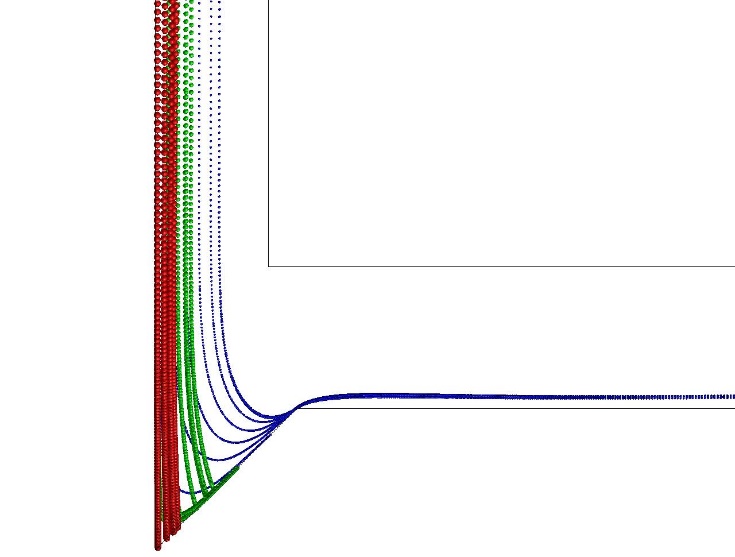 | 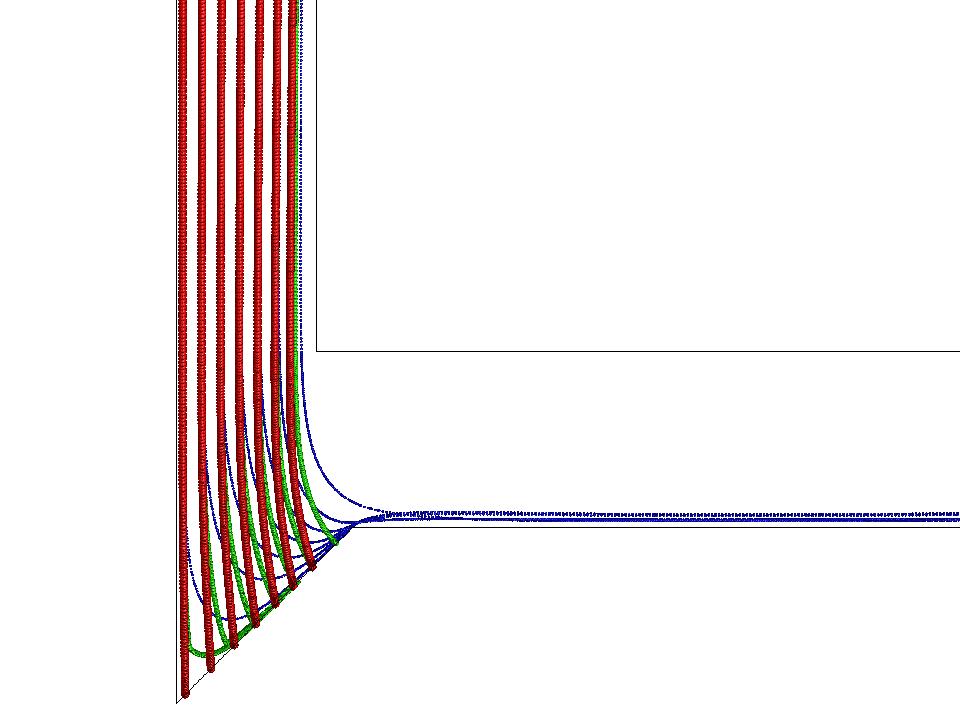 |
| --- | --- | --- |
| a | b | c |
| Figure B. Trajectories of particles in different impactors at 1 slpm. (a) particle trajectories in the AF µ-well impactor. The dashed area is expanded in (b) to show the details; (c) detailed view of the particle trajectories for the µ-well impactor with a straight nozzle. Particle size: red line – 3 µm, green line – 2 µm, blue line – 1 µm (Nozzle Diameter: 0.8mm; Re=1815), from [1] | | |

The micro-well (µ-well) particulate collection cartridge consists of two injection-molded UV-transparent parts. The top half of the cartridge includes an aerodynamic focusing (AF) inlet to accelerate and focus the particles and a big outlet to the vacuum pump. The conical µ-well on the bottom half captures the particles into a small collection area. Figure C shows the conceptual diagram and the drawing of the cartridge with the dimensions of the AF inlet and the µ-well. The diameter of the AF inlet is chosen based on the calculation of the Stokes number, as well as compatibility with the standard pipette tip. The angle of the µ-well cone of 35 degrees is selected to reduce the effect of particle bounce at the collection location.

| 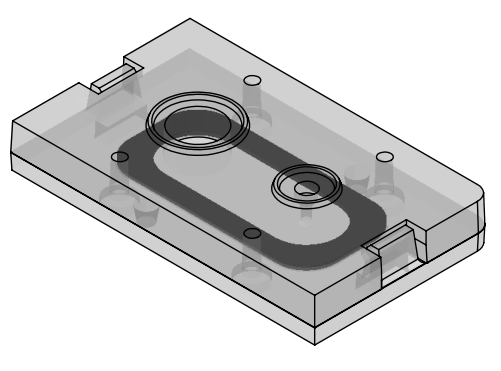 | 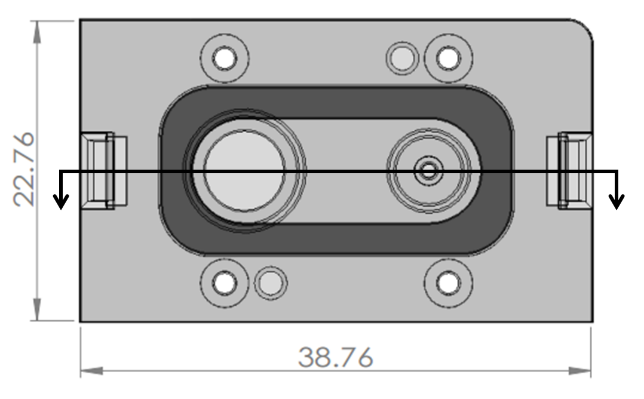 |
| --- | --- |
|  | b |
|  | 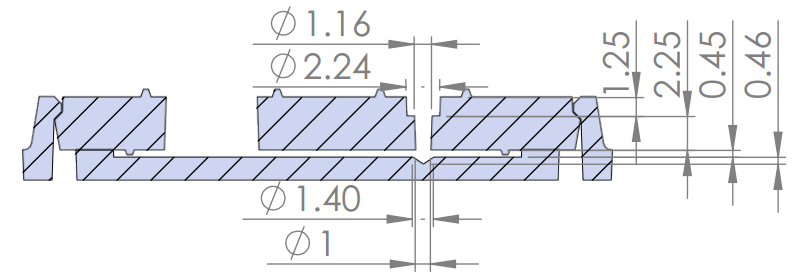 |
| a | c |
| Figure C. (a) The µ-well aerosol collection cartridge and (b) the dimensions of the cartridge; (c) the critical dimensions of the AF inlet and the µ-well (unit: mm) | |

The µ-well cartridges were tested for their usability in a pilot field study, in which the particulate matter (PM) samples were collected from users for the assessment of the health risks related to PM exposure. Figure D illustrates the differences in PM collected for two of the users, with high versus low exposures. This epidemiological study is underway, analysis of the samples will be presented in the future manuscripts.

| **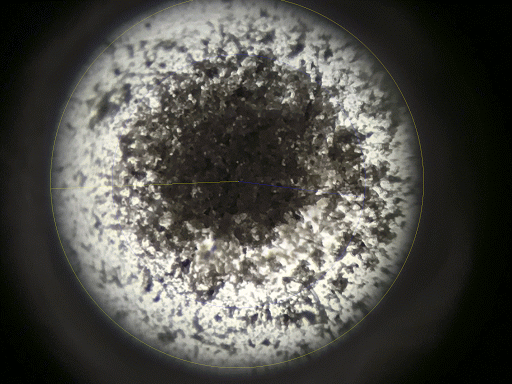** | **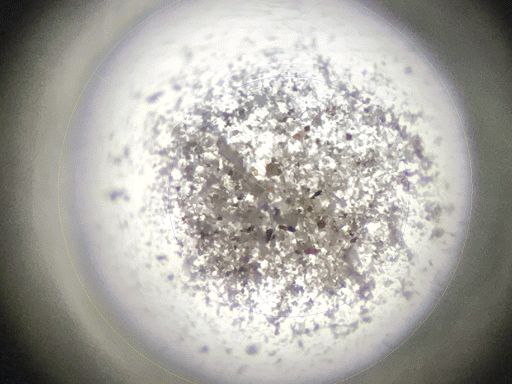** |
| --- | --- |
| Figure D. Microscopic image of PM collected in the cartridges during the one-week usability study. | |

| 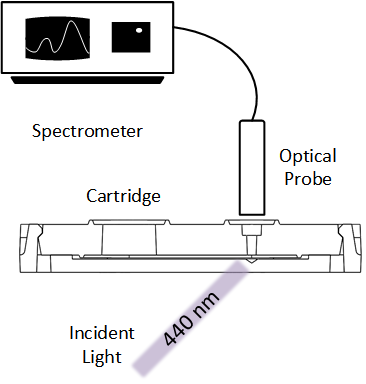 | 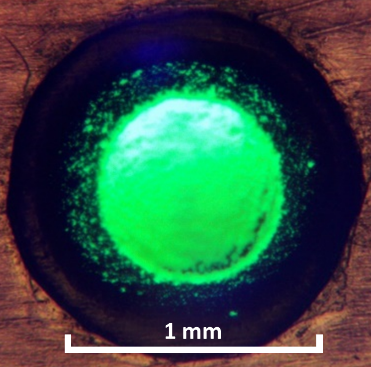 |
| --- | --- |
|  | b |
|  | 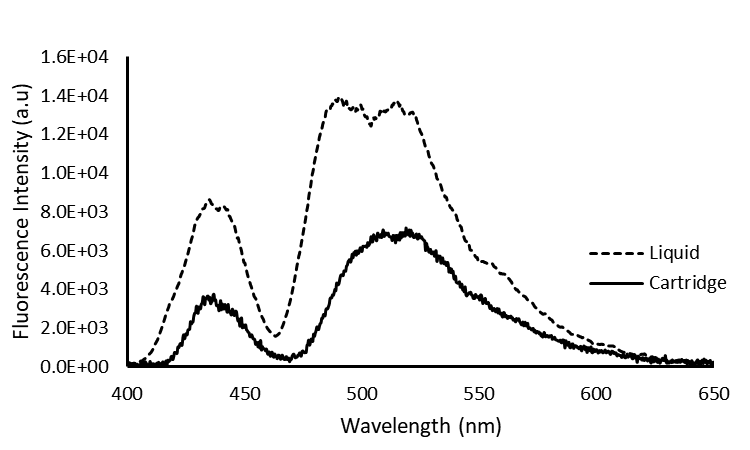 |
| a | c |
| Figure E. (a) The cartridge fluorescence measurement setup and (b) the fluorescent PSL particle collection site; (c) the fluorescence spectrum for the liquid and solid sample. | |

In the proof of concept experiment to test cartridge’s compatibility of the in-situ optical analysis, the fluorescence measurement of polystyrene latex (PSL) microspheres was performed. Figure E shows the schematics of the fluorescence measurement setup and the fluorescence spectrum from the PSL particles. The 2 μm fluorescent particle (Polysciences Inc.) were aerosolized in the hermetically sealed chamber and collected at 1 slpm, which results in a dense cluster of particles as shown in Figure Eb. The sample collected in the µ-well was then excited with the 440 nm light from the bottom at the 45-degree incident angle. The excitation light was generated by passing the light from the DH-2000-BAL deuterium halogen source (Ocean Optics, Dunedin, FL) through a linear variable bandpass filter (Ocean Optics, Dunedin, FL). The Flame NIR spectrometer (Ocean Optics, Dunedin, FL) was used to analyze the emission light from the sample collected through an optical fiber probe placed above the µ-well. The yellow-green PSL particle has the maximum excitation at about 440 nm and the maximum emission at about 490 nm. We compared the fluorescence spectrum acquired from the cartridge sample to the spectrum from the liquid sample measured in a quartz cuvette (see Figure Ec). The liquid sample fluorescence measurement was performed in the CUV-ALL-UV Cuvette Holder (Ocean Optics, Dunedin, FL) at 90-degree angle. Most of the scattering signal from the blank cartridge and the solvent was filtered from the spectra, however, some excitation signal is still apparent in the recorded spectra.

**Reference**

1. He, J. and I.V. Novosselov, *Design and evaluation of an aerodynamic focusing micro-well aerosol collector.* Aerosol Science and Technology, 2017. **51**(9): p. 1016-1026.
